# Empirical study on the impact of tax reduction on the development of Chinese green energy industry

**DOI:** 10.1371/journal.pone.0294875

**Published:** 2023-11-30

**Authors:** Yang Cao, Xu Liu

**Affiliations:** JiLin University of Finance and Economics, Changchun, China; Universiti Malaysia Sabah, MALAYSIA

## Abstract

The implementation of tax reduction policies in China represents a significant and effective strategy. Accordingly, this strategy has been designed to facilitate the development of a green economy by establishing a market-oriented allocation system for environmental and resource elements, while simultaneously invigorating microeconomic entities. As the nation navigates towards the adoption of green, low-carbon production, and lifestyles, the role of clean and green energy emerges as a vital necessity. Therefore, to explain the impact of tax reduction policies on the green energy industry, this study collected and compiled financial indicator data from 100 listed companies in the green energy sector, utilizing the China Stock Market Accounting Research database (CSMAR) as a source for research samples. A Panel Vector Auto Regression (PVAR) model was employed to observe the effects of tax reduction policies on the energy industry, while the dosage effects Difference in Difference (DID) model was utilized to verify and supplement the findings. In summary, the findings of this study can be summarized as follows: firstly, tax reduction policies exert a positive impact on the green energy industry by effectively mitigating the financial cost burden on green energy enterprises, thereby reducing production expenses and amplifying their profitability. Secondly, such policies bolster the capital turnover rate of enterprises in the short term, thereby enabling augmented research and development investments, refining production efficiency, and enhancing competitiveness. Through rigorous analysis and demonstration, the research findings accentuate the stimulative and propulsive impacts of tax reduction policies on the flourishing development of the green energy industry. Furthermore, this study provides relevant fiscal and tax policy recommendations, thoughtfully derived from the research findings.

## 1. Introduction

China, as one of the world’s largest energy consumers and emitters of greenhouse gases, has made active responses in recent years to the challenges posed by climate change. According to the reports of the 20th National Congress of the Communist Party of China, a plan was put forth to improve ’fiscal, taxation, financial, investment, and pricing policies and systems of standards’ in order to support green development. The aim was to boost green and low-carbon industries, as well as to improve the system for market-based allocation of resources and environmental factors. Additionally, there was a plan to accelerate the research and development, promotion, and application of advanced energy-saving and carbon emission reduction technologies, to encourage green consumption, and to promote green and low-carbon ways of production and life. In September 2020, China announced at the 75th United Nations General Assembly that it would increase its national voluntary contributions, strive to peak carbon dioxide emissions by 2030, and make efforts to achieve carbon neutrality by 2060. To achieve "carbon peaking" and "carbon neutrality," promoting the adoption of clean energy production and enhancing energy efficiency and environmental protection in businesses became essential steps. Furthermore, it was crucial for companies to benchmark against both international and domestic advanced standards in these areas. Notably, the country has been making strenuous practical actions to drive its green development.

**Fig 1:**
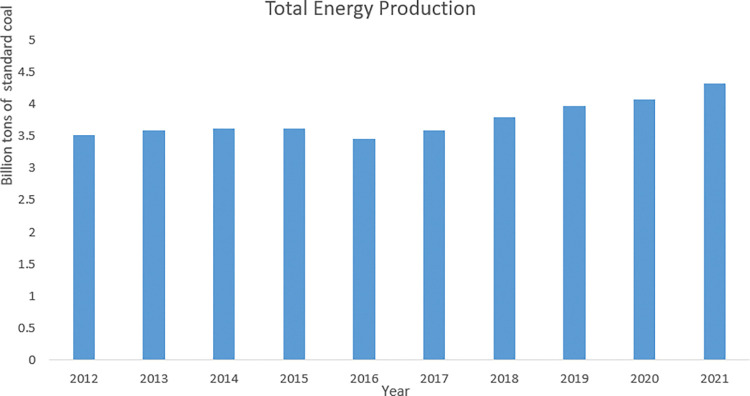
Total energy production in China from 2012 to 2021. Source: China Statistical Yearbook in 2022.

In accordance with the "Statistical Bulletin on National Economic and Social Development in 2022" published by the National Statistics Bureau, China’s proportion of clean energy consumption increased by 0.4 percentage points to 25.9% in 2022, compared to the previous year. Simultaneously, the installed capacity of renewable energy power generation also exhibited growth of 7.8% from the previous year, reaching 2.56 billion kilowatts. Moreover, over the period of 2012 to 2021, China’s overall energy production demonstrated an upward trend, with the total production escalating from 3.51 billion tons of standard coal in 2012 to 4.33 billion tons of standard coal in 2021, attaining the highest production level in almost a decade (see **Fig 1**).

Since 2018, China has implemented a series of inclusive tax cut policies, particularly in late 2019, when the outbreak of the COVID-19 pandemic and frequent lockdowns dealt a heavy blow to many industries, including the tertiary sector, entertainment, and manufacturing. In response, China introduced large-scale tax relief policies, including reduced value-added tax rates and exemptions from corporate income tax. With tremendous investments in epidemic prevention and control, coupled with long-term issues related to local debt, many already outstrecthed local governments are now confronted financial crises. As a result, the economy has ushered in a period marked by sluggish growth (with a GDP growth rate of 3% in 2022, only 0.6 percentage points higher than during the COVID-19 pandemic in 2020) since the post-pandemic era of 2022. The government’s continued loose monetary and fiscal policies have yielded unsatisfactory results. In this context, it is extremely crucial to study how to make positive use of fiscal policies as automatic stabilizers to assist in the steady growth of China’s economy and enhance the incentive and precise targeting effects of tax policies. This also denies one-size-fits-all or indiscriminate policies adopted by the government in their preferential treatment. As the Chinese saying goes, ’good steel used in the blade’ to achieve maximum effectiveness with few resources. Following the analysis, further research remains to be conducted on the role of tax cut as a contributor to green transformation.

In a parallel effort to facilitate a comprehensive green transformation in economic and social development and implement sustainable development strategies, China has implemented 56 tax and fee preferential policies to support green development. Specifically designed to support environmental protection, these policies also promote energy conservation and environmental preservation, encourage the comprehensive utilization of resources, and foster the development of low-carbon industries. In light of efforts to achieve an environmentally friendly economic transformation and reduce energy consumption, the new energy industry has been designated as a strategic pillar for the country’s future [1, 2]. The efficiency of green energy [3], thereby, plays a crucial role in driving the transformation towards a sustainable future [4]. Moreover, Cui Y et al. [5] constructed a differences-in-differences model using industrial firms, pollution, and patent data to investigate the impact and mechanism of tax reduction on firms’ energy efficiency, found that tax reduction can significantly improve firms’ energy efficiency. Therefore, it becomes crucial to focus on effectively implementing fiscal and tax policies to facilitate the robust and stable growth of China’s green energy industry. By developing green energy and advancing clean technologies, we can address the significant dependence on energy imports in the country and mitigate energy wastage [1, 6]. Thus, through the simultaneous promotion of decarbonization, pollution reduction, afforestation, and economic growth, ecological preservation, resource conservation, and optimization can be prioritized, fostering a green and low-carbon development path.

Utilizing data provided by the CSMAR database, this study examines the financial data of 100 carefully selected listed companies in the green energy industry. Moreover, the PVAR model and the dosage effects DID model are employed for analysis. Subsequently, the study reveals several key findings: (i) a reduction in tax burden leads to a decrease in the financial expense ratio of companies, thereby enhancing their profitability; (ii) the decrease in tax burden has a positive impact on the capital accumulation rate of companies, demonstrating a certain level of sustainability; (iii) the reduction in tax burden has a temporary positive effect on the cash ratio of companies. However, as the policy is implemented, this effect gradually transitions to a negative impact, resulting in a decrease in the debt-paying ability of green energy companies; and (iv) the reduction in tax burden has a fluctuating impact on the turnover rate of current assets of companies. Generally speaking, it has a relatively positive effect on their operational capacity.

The innovation of this study is as follows: (i) Tax incentive policies are adopted by the government to promote economic growth and adjusts industrial structure. China, to fulfil its responsibilities as a major nation, has introduced tax preferential policies for green development, especially in the green energy industry. However, there remains limited research on the impact of taxation on the development of this industry. This direction represents an innovative perspective in the research question; (ii) In 2019, the PVAR model was applied for the first time [7] to analyze the impact of tax cuts on the four major financial indicators of listed companies in the financial industry. To further verify its feasibility, this study combines the dosage effect DID model with the PVAR model to examine the impact of tax cut policies on the green energy industry. The two methods exhibit mutual corroboration, yield universally anticipated conclusions in qualitative aspects, and produce encouraging results in quantitative and short- to long-term effects analysis. This represents a methodological innovation. Furthermore, the method is applicable to the analysis of tax preferential effects across all industries, with replicability and generalizability. It can be employed for cross-sectional comparisons to measure the heterogeneous impact of tax incentives on enterprises in different industries.

## 2. Literature review and research hypotheses

### 2.1 Literature review

On May 1, 2016, China began to fully implement the policy of replacing business tax with value-added tax. Since the policy was previously piloted in Shanghai, Beijing, Shenzhen and other places, with experimental groups and control groups, a large number of literatures on the effect analysis of the policy of replacing business tax with value-added tax emerged during this period. Fang H et al. [8] found that the replacement of business tax with value-added tax has significantly reduced the total tax burden of small-scale taxpayers, and there is no heterogeneity between the transportation industry and the modern service industry. Peng F et al. [9] concluded that the business tax reform has a positive impact on the total factor productivity of manufacturing enterprises, and simplifying the tax system in different industries helps to improve the productivity of enterprises. Yu J, Qi Y [10] studied the impact of changing business tax to value-added tax on productivity. The results showed that this reform has increased the productivity of enterprises by 14.6% on average.

The macroeconomic effects of tax reduction and fee reduction are quite evident, bringing about positive impacts at the micro-level on corporate profitability, innovation research, etc. As the value-added tax reform has been in progress, the academic community has gradually shifted its focus to the study of the effects of cutting taxes and administrative fees. From a macroeconomic perspective, tax reduction promotes economic growth [11, 12] and reduces the tax burden for most industries, including the real economy [13]. Njogu LK [14] used Kenyan national data to conclude that there is a significant negative correlation between the value-added tax rate and gross domestic product. Y Neog,AK Gaur [15] used the data of 14 states in India to analyze the relationship between taxation and economic growth, and concluded that income tax and commodity service tax have a negative impact on the economic growth of each state.

At the micro level, the difference-in-differences (DID) or regression methods are mainly used to obtain the impact of tax cuts on corporate profitability [16, 17], innovative R&D [18–20], fixed asset investment [21, 22], enterprise access [23]; there are positive correlation conclusions, and there are certain industry heterogeneity and regional heterogeneity. Mukherjee A et al. [24] argued that with the increased implementation of tax cuts and the reduction in the level of tax burden on firms, firms have more disposable income to invest in R&D and innovation activities and the level of innovation at the micro level is developed. Ohrn E [25] analyzed the impact of accelerated depreciation policy on the U.S. manufacturing industry by constructing a modified difference-in-difference framework, and the results showed that the implementation of accelerated depreciation policy has a significant impact on capital investment, this impact is somewhat volatile.

While tax reductions bring about several positive outcomes, it’s important to acknowledge that they can also have adverse fiscal consequences. As such, it is imperative to formulate optimal tax reduction strategies from a policy perspective. This entails taking a macroeconomic view to assess the impact of tax reductions on government fiscal revenues [26, 27], local debt risks [28], and the sustainability of tax and fee reductions [29, 30]. Liu Q, Zhang X [31] analyzed the impact of tax reduction policies on fiscal sustainability by collecting panel data from different provinces in China through the FGLS empirical method. The results showed that the implementation of tax reduction policies can have a negative impact on fiscal sustainability. Tax cuts can have a significant dampening effect on fiscal revenues while promoting economic growth, and the effect on fiscal revenues can vary across countries depending on the pattern of tax cuts implemented [32].

Reducing corporate tax rates help foster a healthy and fair competitive environment for businesses [33], stimulate the economy growth. However, it can also increase the fiscal deficit, which potentially leads to a fiscal crisis [34]. Striking a balance between severe fiscal pressures and, large-scale tax and fee reduction to benefit the people is essential. This process can pave the way for a broader discussion on optimizing China’s medium and long-term tax system.

### 2.2 Research hypotheses

Theories and principles related to taxation and fixed costs assert that taxation imposes fixed costs on enterprises. Given that tax policies can significantly impact financial health, for instance, with respect to the payment of fixed costs. A weightier tax burden may potentially erode a company’s profitability, rendering it more challenging to meet its fixed cost commitments, consequently leading to financial strain. In this paper, we formulate these assumptions for discussion and conduct a systematic investigation of each one. Klapper LF, Tzioumis K [35] argued that if a firm bears a lower tax burden, it can increase its equity and reduce its business risk, thus improving its performance. Wagner AF et al. [36] suggested that implementing tax cuts for firms with a higher tax burden can lead to more benefits. Ma J et al. [37] used the difference-in-difference (DID) method with data covering 22 listed companies from 2009 to 2020, and the empirical study showed that BT to VAT reduces the tax burden of companies. Isik S, Özbuğday FC [38] tested whether tax cuts would reduce fertilizer prices by DID and the results showed that consumers would benefit from the tax cuts. Zhang S [39] constructed a DID model using data of all A-share listed companies in China from 2009–2015, the study showed that tax reduction can directly reduce the tax burden of enterprises and promote the development of the real economy.

On this basis, this paper posits:

**Hypothesis 1: Tax cuts and fee reduction policies will alleviate the tax burden on green energy enterprises to enhance their profitability**

In accordance with Tax Incidence Theory, within various tax structures, taxes can play a role in either promoting or hindering growth for businesses. For instance, double taxation might impede investments and employment, thereby impacting industrial development. Based on Ability-to-Pay Theory of Taxation, faced with tax increments, companies may reduce consumption and output. Drawing upon the Welfare Theory, with rising taxes, companies may shift expenditures from goods and services to alternative forms of consumption, conceivably leading to a decline in production. These examples illustrate the specific responses that businesses might have to tax increments. Meanwhile, fluctuations in the tax burden can significantly influence industry development. Therefore, diverse methodologies have been adopted to study the impact of tax reductions on businesses. Manda S, Bansal SK [40] believed that the potential positive economic outcomes of corporate tax cuts outweigh the negative ones, and that corporate tax cuts will help improve India’s competitiveness and cost of doing business in the global marketplace. Zhang H [13] concluded through his study that the real economy is getting better under the effect of tax reduction and fee reduction. Gechert S, Heimberger P [41] applied meta-regression methods to a new dataset and the results showed that corporate tax cuts promote economic growth.

On this basis, this paper posits:

**Hypothesis 2: Tax cuts and fee reduction policies can enhance the short-term development capacity of the green energy industry.**

## 3. Methodology

### 3.1 PVAR

Panel Vector Auto Regression Model (PVAR) was first proposed by Holtz Eakin et al. [42]. The PVAR model is characterized by a large cross-section and a short time series, and is able to address individual heterogeneity through the use of panel data, taking into account both individual and time effects.

Because the data of green energy enterprises is 26 quarterly index data of 100 enterprises, it belongs to short-term panel data. In order to capture the dynamic interaction between endogenous variables in panel data and effectively solve the problems of endogenous and individual heterogeneity in panel data, it is appropriate to use panel vector autoregressive (PVAR) model. Through Eviews10.0 and Stata17.0 software, the specific PVAR model formula is as follows:

$$y_{it}=\alpha_{0t}+\alpha_{1}y_{it-1}+\cdots +\alpha_{p}y_{it-p}+f_{i}+\theta_{t}+\varepsilon_{it}\;i=1,2,\ldots ,N;t=1,2,\ldots ,T$$ (1)

In the above equation, i represents different listed companies, t represents different quarters, *y*_*t*_ is a vector of columns of endogenous variables containing the four major capability indicators of financial analysis and tax burden, p stands for the lag order, *γ*_*it*_ represents the perturbation column, *f*_*i*_ represents the individual effect, *ϵ*_*t*_ represents the time effect, and it is estimated using the generalized moment method(GMM).

### 3.2 Dosage effects DID

The traditional differences-in-differences model requires that a part of the sample is interfered by the policy, that is the treatment group, while the other part of the sample is not interfered by the policy, that is the control group. However, when the current tax reduction policy is issued, it is uniformly implemented nationwide, that is, the introduction of the policy will have an impact on the corresponding individuals nationwide, which makes it difficult to define the treatment group and control group of the traditional differences-in-differences model. The DID model with dosage effects can well solve the problem that the traditional DID is difficult to determine the treatment group and the control group under the national one-size-fits-all policy. The dosage effects can divide the samples according to the degree of systematic influence, and realize the construction of the control group and the experimental group in this way. On the basis of using the dose effect to divide the samples into the treatment group and the control group, the DID model is used to empirically test the treatment group and the control group, so as to realize the effect evaluation of the national one-size-fits-all policy. During the experiment, in order to reduce the impact of individual and time differences on the experimental results, this paper controls the individual effect and time effect of the sample when evaluating the effect of tax reduction policy through the DID model. The specific formula is as follows:

$$y_{it}=\beta_{0}+\beta_{1}{Treat}_{i}*{Post}_{t}+\beta_{2}{Treat}_{i}+\beta_{3}{Post}_{t}+\beta_{4}X_{i,t}+\delta_{i}+\gamma_{t}+\varepsilon_{it}$$ (2)

In the above equation, i represents the enterprise, t represents the time, *y*_*it*_ is the explained variable, and *Treat*_*i*_ * *Post*_*t*_ is the explanatory variable, that is, the cross-multiplication term of policy implementation, which is the dummy variable that the first i enterprise is affected by the policy of replacing business tax with value-added tax in the t year; since the time for China to implement the comprehensive policy of replacing business tax with VAT is the second quarter of 2016, when dividing the tax burden level, this paper divides the sample into three equal parts according to the average comprehensive tax rate of green energy enterprises in the first two years of policy implementation, that is, from 2014 to 2015; *Treat*_*i*_ is the indicator variable. When it is the treatment group, that is, the tax burden level of the enterprise is in the highest 1 / 3 group, the value of the variable is 1. When it is the control group, that is, the tax burden level of the enterprise is in the lowest 1 / 3 group, the value of the variable is 0. *Post*_*t*_ is the time effect of policy implementation, *Post*_*t*_ is equal to 0 when the time is before the second quarter of 2016, and *Post*_*t*_ is equal to 1 when the time is after the second quarter of 2016. *X*_*i*,*t*_ is the control variable, *δ*_*i*_ is the individual fixed effect, *γ*_*t*_ is the time fixed effect, and *ε*_*it*_ is the error term.

## 4. Index selection and variable setting

### 4.1 Data sources

Through CSMAR database, this paper selects and collects the data of four financial indicators of profitability, growth ability, solvency and operating capability. In order to balance the data, quarterly data are used. The selected data interval is from Q2 2016 to Q3 2022, but excluding companies listed after Q2 2016 as well as ST and *ST listed companies, 100 listed companies were selected after exclusion, and each company contains 26 quarterly of data.

### 4.2 Index selection and variable setting

The index selection method refers to the method in Cao Y et al. [7], and screens and classifies the collected data. In order to ensure the integrity and accuracy of the results, the indicators with serious data loss are screened out, and 14 variables are finally obtained by calculation, including 4 financial indicators and 1 tax burden index. The following are the four major financial indicators of listed companies selected from CSMAR database. Among them, the profitability indicators are: operating cost rate (x1), sales cost rate (x2), management cost rate (x3), financial cost rate (x4); the growth ability indicators are: capital accumulation rate (x5), total asset growth rate (x6), owner equity growth rate (x7); the solvency indicators are: current ratio (x8), cash ratio (x9), asset-liability ratio (x10); the operating capacity indicators are: total assets turnover ratio (x11), turnover of current assets (x12); the tax burden indicators are the comprehensive tax rate A and the comprehensive tax rate B (x13, x14).

#### 4.2.1 Stationarity test

Due to the time series nature of panel data, it is vital to test the stationarity of each sequence in order to make the data more accurately explain the problem and prevent ’spurious regression ’.This paper adopts the LLC and ADF tests. The following **Table 1** lists the results of the ADF test. The results show that at the significance level of 1%, the P values corresponding to the unit root test values of the three test methods are all less than 0.01, so the null hypothesis is rejected, which indicates that all variables are stationary sequences.

**Table 1:** Stability test results of each index.

Variable	ADF-Fisher	Variable	ADF-Fisher
**X1**	356.140 (0.00)	**X8**	356.522 (0.00)
**X2**	370.522 (0.00)	**X9**	605.533 (0.00)
**X3**	396.816 (0.00)	**X10**	517.927 (0.00)
**X4**	424.346 (0.00)	**X11**	282.368 (0.00)
**X5**	723.720 (0.00)	**X12**	339.880 (0.00)
**X6**	590.458 (0.00)	**X13**	28.656 (0.00)
**X7**	723.700 (0.00)	**X14**	43.879 (0.00)

#### 4.2.2 Correlation test

The correlation test of the above variables is carried out to improve the accuracy of the model estimation. In this paper, Eviews10.0 software is used to eliminate the variables with correlation, and a variable is selected from each index as a representative. There is a significant correlation between the variables at the level of more than 10%. Among them, X13 and X14 are tax burden indicators, but X13 is related to most of the selected indicators, so X13 is eliminated and X14 is retained. At the same time, the tax burden index is the focus of this paper, and the variables must be retained. In terms of development ability and solvency, compared with other indicators, X5 and X9 are related to fewer variables. Therefore, capital accumulation rate (X5) and cash ratio (X9) are selected as representatives of development ability and solvency indicators.

The variables related to X5, X9 and tax burden indicators need to be eliminated, so the current asset turnover rate (X12) is selected as the representative in the operating capacity, and X1, X2 and X3 related to X12 need to be eliminated, so the financial expense rate (X4) is selected as the representative of profitability. In summary, five variables are selected from 14 variables: financial cost rate, capital accumulation rate, cash ratio, current asset turnover rate, and comprehensive tax rate B, which are recorded as Y1, Y2, Y3, Y4 and Y5 as variables of PVAR model, as shown in the following **Table 2**.

**Table 2:** Description of related data variables.

Financial index	Variable name	Variable	Computing formula
Profitability indicators	Financial cost rate	Y1	Financial expenses / operating income
Growth ability index	Rate of capital accumulation	Y2	(The end of the current period of total owner ’s equity-the initial value of the current period of total owner ’s equity) / the initial value of the current period of total owner ’s equity
Indicators of solvency	Cash ratio	Y3	Cash and cash equivalents closing balance / current liabilities
Operational capability index	Turnover of current assets	Y4	Operating income / current assets closing balance
Tax burden index	Comprehensive tax burden	Y5	(Business tax and additional + income tax expenses) / total profit

## 5. Empirical analysis and conclusions

### 5.1 Pvar model result

**Fig 2:**
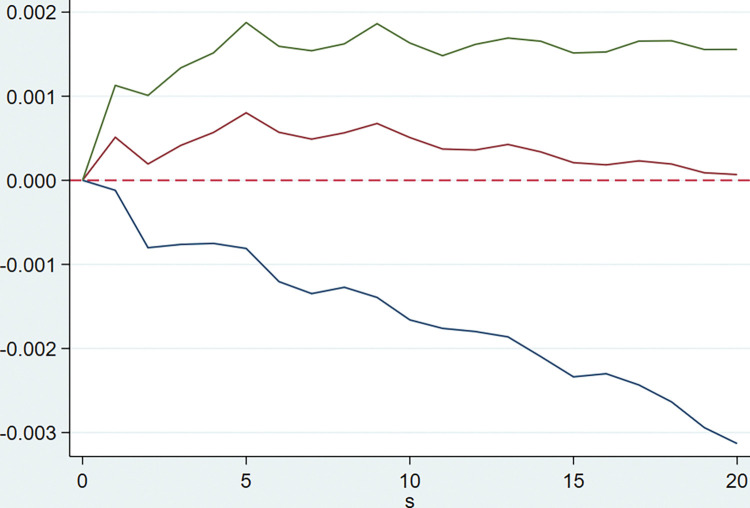
Response of financial cost rate to the tax burden.

**Fig 3:**
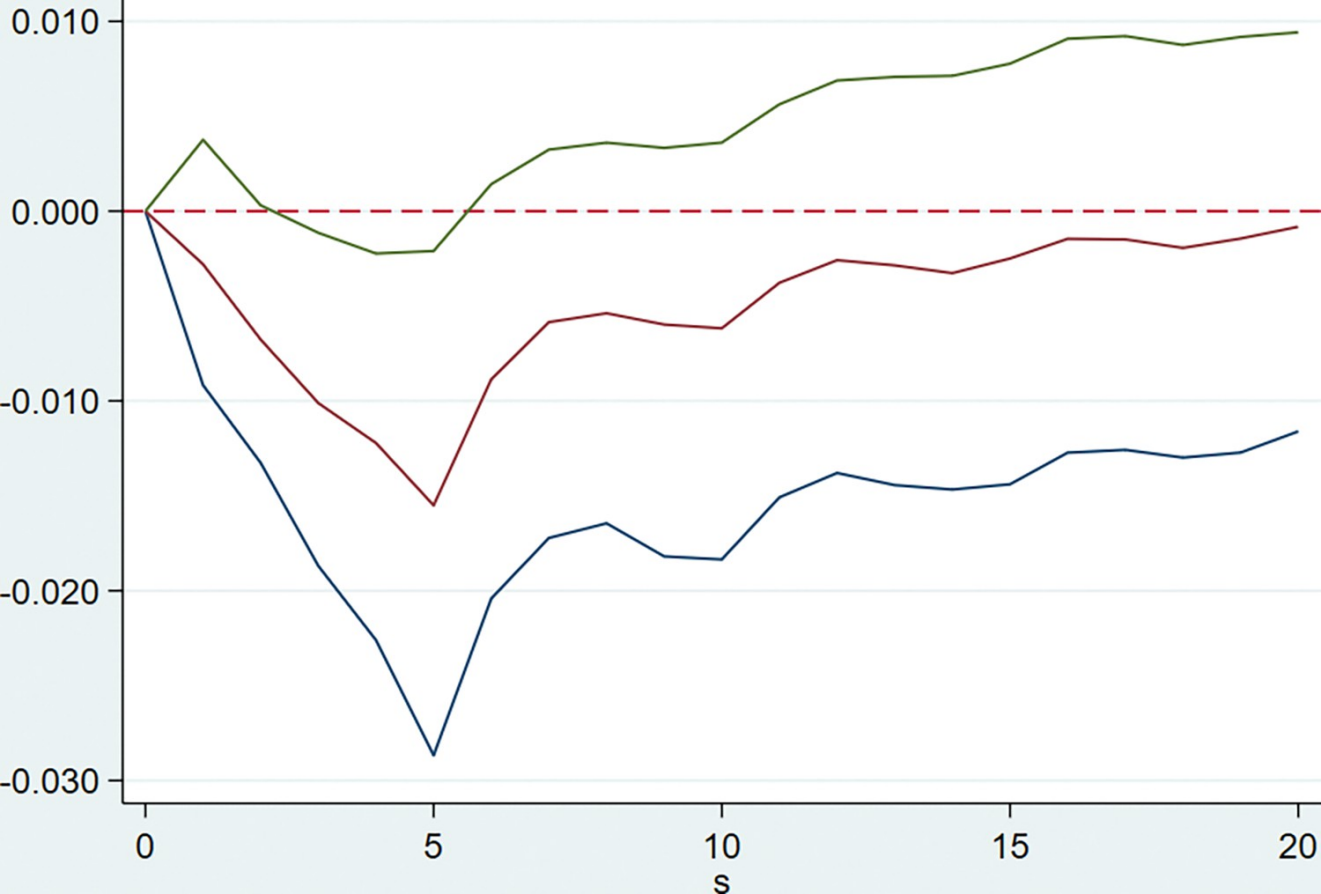
Response of rate of capital accumulation to the tax burden.

**Fig 4:**
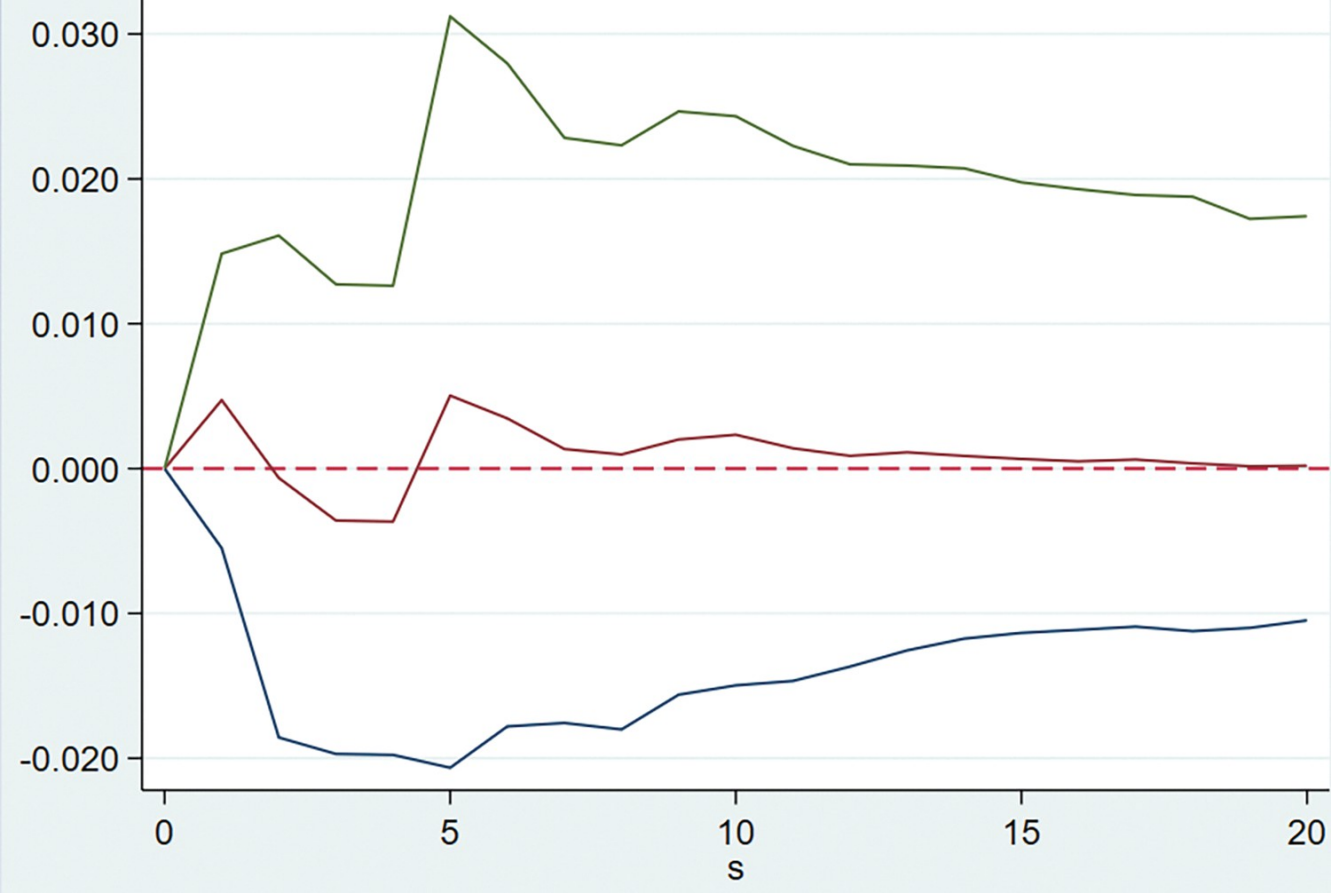
Response of cash ratio to the tax burden.

**Fig 5:**
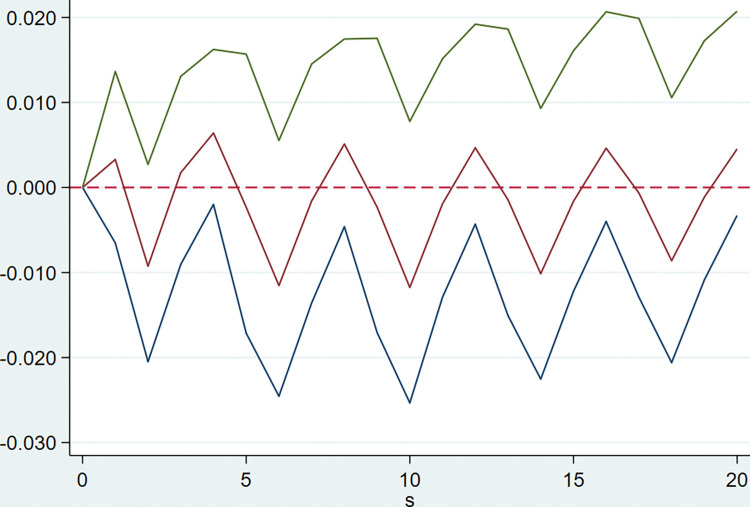
Response of turnover of current assets to the tax burden.

The model is implemented by Stata software using the PVAR2 package provided by Lian Y. **Table 3** shows that the optimal lag order of the model AIC, BIC and HQIC values are all of order 5, that is five quarterly lags. After that, the PVAR model is established, and the GMM estimation of the model is carried out. The following impulse response Figs 2–5 are obtained for the tax rate.

**Table 3:** Optimal lag order estimation for PVAR models.

Lag	AIC	BIC	HQIC
**1**	0.465445	1.69012	0.910095
**2**	-0.378598	0.94947	0.104615
**3**	-0.741931	0.69737	-0.217087
**4**	-2.83255	-1.27319	-2.26261
**5**	-3.47231*	-1.78357*	-2.8536*

In the figure above, the horizontal axis represents the number of periods, and the vertical axis represents the fluctuation caused by the unit shock. **Fig 2** represents the dynamic impact of tax burden on the financial expense ratio, which shows that when the tax burden is subjected to external shocks, it will have a longer-term positive promotion effect on the financial expense ratio. This demonstrates that the financial expense ratio of businesses will decrease when the overall tax burden declines, that is, the implementation of the tax reduction policy will reduce the tax burden of listed companies, reduce the expenses of enterprises, and help to improve the profitability of listed companies.

**Fig 3** represents the dynamic impact of the tax burden on the capital accumulation rate, when the overall tax burden is hit by one standard deviation, there will be a sustained negative impact on the capital accumulation rate, and then gradually converge to 0. This suggests that if the overall tax rate is lowered, it will promote the growth of the capital accumulation rate of the enterprise, and it will be a long term impact, which is beneficial to the long term development of the enterprise.

**Fig 4** represents the dynamic impact of tax burden on cash ratio, it can be seen that when the comprehensive tax burden is subject to external shocks, the year-on-year growth rate of the cash ratio first rises, and begins to decline around the second quarter, this negative impact reaches its maximum in four quarters, and then the negative effect gradually diminishes and converts into a positive effect, and the positive effect gradually converges to zero in six quarters. This suggests that, if the current This suggests that if the current combined tax rate were to fall, the cash ratio would first fall in the next quarter, then start to improve in about two quarters, and then gradually fall to 0.

**Fig 5** represents the dynamic impact of tax burden on current asset turnover, it can be seen that when the tax burden is subjected to a positive shock of one unit, the results are more volatile between ± 0.1, accordingly, it is considered that when the combined tax rate decreases, it brings insignificant impact on current asset turnover.

### 5.2 Dosage effects DID model result

As a commonly used econometric model to test the policy effect, the DID model plays a very important role in testing the policy effect. To demonstrate the impact of tax cuts on the development of green energy companies, this paper makes a confirmatory test on the above results through the DID model. Because the tax reduction policy is a nationally unified policy, it cannot meet the requirements of the traditional Differences-in-Differences model for the treated group and the control group. Based on the research methods of Qian X et al. [43], this paper divides the samples by using the dosage effect to create the control group and the treated group. The common dose effect score methods are dichotomy, trisection and quartile. Since the sample used in this paper is 93 listed A-share green energy companies, to ensure the accuracy of the empirical results, the trisection method will be used for testing. At the same time, this paper selects enterprise size and sales volume as control variables. Among them, the enterprise size selects the total assets data of the enterprise for measurement, and the sales volume selects the business income data of the enterprise for measurement, and standardizes the data. In the empirical results, we mainly focus on the regression coefficient *β*_1_ of *Treat*_*i*_ * *Post*_*t*_, which measures the DID effect of tax reduction on enterprises.

#### 5.2.1 Descriptive statistics

In order to overcome the influence of extreme values on the empirical results, this paper performs Winsorize tail reduction on all continuous variables at 1% and 99% quantiles, and uses Stata17.0 software for data processing. Descriptive statistics are made on the financial index variables of the sample enterprises, and the results are shown in the following **Table 4**. It can be seen from the results in the table that the total sample size is 3627, of which the maximum comprehensive tax burden is 2.705 and the minimum is − 1.389. The gap between the two is large, indicating that there is a certain gap in the actual tax burden between enterprises.

**Table 4:** Descriptive statistical results.

Var Name	Obs	Mean	SD	Min	Median	Max
Financial Cost Rate	3627	0.041	0.061	-0.030	0.022	0.368
Rate of Capital Accumulation	3623	0.071	0.184	-0.320	0.025	1.020
Cash Ratio	3627	0.407	0.491	0.029	0.255	3.263
Turnover of Current Assets	3627	0.647	0.527	0.051	0.505	2.900
Tax burden	3626	0.284	0.468	-1.389	0.225	2.705
Enterprise Size	3627	0.044	0.072	0.001	0.018	0.428
Sales	3627	0.038	0.069	0.000	0.014	0.457

#### 5.2.2 Trisection method

Implementation of tax cuts effectively reduces the tax burden on the green energy sector, but the tax burden of different green energy enterprises is different. Therefore, this paper divides the samples into three equal parts according to the average comprehensive tax rate of green energy enterprises from 2014 to 2015. Since the tax reduction effect of green energy enterprises with higher tax burden before the policy is more obvious than that of green energy enterprises with lower tax burden before the policy, 1/3 of the higher tax burden is taken as the treated group, and the corresponding Treat1 is 1. The 1/3 of the lower tax burden is used as the control group, and the corresponding Treat1 is 0. To ensure the accuracy of the experimental results, this study controls the influence of individual effect and time effect. **Table 5** shows the relevant results. Among them, the column titled Y1 represents the profitability index, the column titled Y2 represents the development ability index, “es” and “sales” are the control variables added (the same below). From the table, according to the cross-multiplication coefficient of Treat and post, the implementation of the tax reduction policy has significantly improved the profitability and development ability of green energy enterprises.

**Table 5:** The treatment effect of high tax burden policy by trichotomy.

	(1)	(2)	(3)	(4)
	a1	a2	a3	a4
VARIABLES	Y1	Y2	Y3	Y4
Treat1_post	-0.0138***	0.1466***	0.1106***	-0.0158
	(0.0029)	(0.0543)	(0.0369)	(0.0348)
es	0.0145	0.8224*	0.4497	-0.9094***
	(0.0262)	(0.4902)	(0.3334)	(0.3138)
sales	-0.0299	-0.4236	0.1568	3.9759***
	(0.0241)	(0.4522)	(0.3076)	(0.2895)
Observations	1,116	1,116	1,116	1,116
R-squared	0.8794	0.1980	0.4494	0.7978

#### 5.2.3 Robustness test

In order to verify the conclusion of DID in the above trisection method, this paper divides the data into three groups by the trisection method, and selects the group with medium tax burden as the treated group, the corresponding Traet2 is 1, the group with the lowest tax burden as the control group, and the corresponding Treat2 is 0. The corresponding results are shown in **Table 6**:

**Table 6:** The treatment effect of medium tax burden policy by trichotomy.

	(1)	(2)	(3)	(4)
	b1	b2	b3	b4
VARIABLES	Y1	Y2	Y3	Y4
Treat2_post	-0.0026	-0.1368	-0.3011**	-0.0389
	(0.0048)	(0.0904)	(0.1256)	(0.0442)
es	0.0531	0.9135	0.4390	-2.2708***
	(0.0457)	(0.8651)	(1.2036)	(0.4231)
sales	-0.2669***	1.3610	1.2382	2.4032***
	(0.0501)	(0.9511)	(1.3207)	(0.4643)
Observations	1,116	1,112	1,116	1,116
R-squared	0.6755	0.1240	0.7923	0.7352

It can be seen from the table that the effect of the implementation of the tax reduction policy on the business activities of green energy enterprises with medium tax burden is the same as that of the three-point high tax policy treatment and the PVAR model, that is, it has little impact on the business activities of green energy enterprises with medium tax burden. Among them, the influence coefficient on profitability and development ability is lower than that of the three-point method when dealing with the high tax burden policy, indicating that the results are robust.

#### 5.2.4 Placebo test

In order to test whether the impact of green energy enterprises comes from the tax reduction policy, the sample is divided into three equal parts according to the comprehensive tax rate of green energy enterprises from 2014 to 2015.If the sample data is before the second quarter of 2015, Post is 0; If the sample data is dated between the second quarter of 2015 and the second quarter of 2016, Post is 1. The resulting DID test results are shown in **Table 7**. From the table, it can be seen that the cross-multiplication coefficient of profitability and development ability is not significant. Compared with the results of Table 5, it shows that the impact of green energy enterprises does come from tax reduction policies, which is the same as the results of the PVAR model.

**Table 7:** Results of placebo test.

	(1)	(2)	(3)	(4)
	c1	c2	c3	c4
VARIABLES	Y1	Y2	Y3	Y4
Treat3_post	-0.0039	-0.0322	0.2427***	-0.0396
	(0.0035)	(0.0264)	(0.0511)	(0.0374)
es	0.5140***	1.3280	1.9901	-10.0210***
	(0.1215)	(0.9183)	(1.7757)	(1.3016)
sales	-0.1089***	0.1918	0.9878*	5.1817***
	(0.0384)	(0.2901)	(0.5609)	(0.4112)
Observations	806	806	806	806
R-squared	0.8908	0.2376	0.5815	0.8256

In summary, the results of the DID test of the sample data through the dosage effects DID model are basically the same as the results of the PVAR model test, which also confirms the reliability of the empirical results of the PVAR model.

## 6. Discussion

This study collected financial indicators data from a sample of 100 listed companies operating in the green energy sector. Empirical research was then conducted utilizing the PVAR model and the dosage effects DID model. The obtained findings provide evidence that tax reduction policies have a significant impact on alleviating the tax burden faced by micro-enterprises, thereby fostering the profitability and growth of green energy enterprises. These findings are consistent with prior research, which has demonstrated that tax reduction policies positively influence profitability, innovation, and research and development activities at the micro-level. Notably, fiscal policy exhibits a more pronounced effectiveness compared to monetary policy, with tax reduction policies outperforming increased fiscal expenditure. It is worth mentioning that since 2016, the Chinese government has emphasized the importance of tax reduction and fee reduction in its annual government work reports, highlighting it as a significant achievement. By 2022, the cumulative tax reduction in China had reached an impressive amount of 12 trillion RMB, equivalent to 11% of the total tax revenue. This process has effectively alleviated the macroeconomic and industry-specific tax burden, yielding tangible benefits for the populace. However, in the face of ongoing developmental challenges, the government has occasionally exhibited a tendency to view tax cuts and fee reductions as a panacea. Therefore, the focus on the development of specific industries or regions has often shifted towards fiscal policy, particularly emphasizing tax reduction and fee reduction measures. This approach has led to a broad application of tax cuts, resulting in reduced marginal benefits. Ultimately, the significance of the policy becomes diluted; resultantly, the formation of a harmonious relationship among tax cuts, increased expenditure, and government debt control becomes unattainable, leading to systemic risks associated with government debt and a lack of perceived benefits for market entities.

Based on the extant body of research and prior work, it is posited that tax reductions elicit varying effects across diverse sectors, geographical regions, and enterprises of varying magnitudes. Throughout the course of our longitudinal research process, we were confined to data from listed companies for the research on the effects of tax reduction due to limited data availability, constituting a limitation of this study. The quartet of major financial indicators assumes a pivotal role in evaluating a company’s fiscal health and operational efficacy. However, it remains a challenging task to select representative indicators from multiple options. In this process, numerous attempts were undertaken to circumvent the curse of dimensionality. For instance, the entropy method was employed to involve the entirety of information in the indicators and synthesize them into a composite index. Nevertheless, weight distortions appeared to have led to ineffective outcomes. To ensure the representativeness of the selected indicators, diverse combinations of specific indicators in the four principal financial indicators of enterprises were subjected to experimentation. However, it is necessary to acknowledge the potential influence of inter-variable correlations on the results. In the empirical process employing the PVAR model, the determination of an optimal lag order primarily relies on information criteria. Therefore, it is necessary to avert the impact of an excessively large lag order on the estimated efficacy of the model parameter. This, in turn, imposes certain limitations on the process of selecting specific indicators.

In response to each potential concern, we have undertaken various efforts and devised a comprehensive methodology for indicator selection. The details of this methodology will be presented in a monograph for publication next year. The empirical methods align with the requisite data type, application scenarios, preconditions, and testing, thereby yielding the anticipated outcomes. Therefore, we assert the justifiability of employing the model.

Premised on long-term research, it is posited that the adherence to the COMB Theory is evident in the implementation of tax reduction measures. Such measures have been observed to contribute to economic growth (smooth hair) along with losses (hair loss). The extent of these losses varies across different industries, owing to their unique characteristics. It is necessary that the teeth of the comb strike a balance between being neither excessively sparse nor dense (which represents excessive and frequent tax incentives). An excessively sparse arrangement renders it ineffective, whereas a greater density leads to significant hair loss (burdensome fiscal responsibility). This phenomenon serves as the underlying reason for the heterogeneity observed in tax policies and highlights the significance of our research. Future socialist tax policies, characterized by Chinese attributes, must be firmly grounded in the Chinese context while embracing a global perspective. The reduction of taxes and fees should be geared towards facilitating the modernization process in line with the Chinese model. Moving forward, it is crucial to conduct a comprehensive analysis of the differential effects of tax reduction on various industries, thereby enabling the implementation of targeted policies. Specifically, targeted tax cut policies should be devised for industries that receive state support, such as the green energy sector. Conversely, for industries characterized by high pollution and energy consumption, it may be appropriate to impose a proportionate increase in tax burden, thereby facilitating the upgrading of the industrial structure.

## 7. Conclusions and policy recommendations

### 7.1 Conclusions

This study employs panel data from publicly traded firms in China’s green energy sector, spanning from Q2 2016 to Q3 2022. Through the utilization of the PVAR model and the dose-response DID model, the study investigates the dynamic effects of the tax burden on the financial expense ratio, capital accumulation rate, cash ratio, and turnover rate of current assets. The results of the study are shown below:

In terms of the profitability of publicly traded firms, the results indicate that the response of the financial expense ratio to changes in the tax burden is always negative. This implies that there is an inverse movement relationship between tax cuts and company profitability. More specifically, a significant reduction in the tax burden leads to a decrease in cost expenses, an increase in profits, and an improvement in the profitability of enterprises, thereby supporting Hypothesis 1.

With respect to the developmental capacity of publicly traded firms, the study finds that reducing the tax burden has a persistent and favorable long-term effect, particularly for companies with a high tax burden. This indicates that tax reduction policies significantly enhance the developmental capacity of enterprises, fostering company growth and supporting Hypothesis 2.

Regarding the debt-paying ability of publicly traded firms, the impulse response graph reveals a fluctuating impact of reducing the tax burden, which tends to converge to zero in the long run. However, the results of the dosage effects DID model indicate that this effect is not statistically significant, suggesting that reducing the tax burden weakens the debt-paying ability of enterprises. This implies that companies with higher tax burdens have relatively lower liquidity, leading to a decrease in their debt-paying ability. To expand their development scale, listed companies may increase their loan amounts from banks.

With respect to the operational capacity of publicly traded firms, the result indicates that the impact of tax burden reduction on the turnover rate of current assets displays some volatility, suggesting no significant correlation between tax reduction policies and the operational capacity of enterprises.

### 7.2 Policy recommendations

#### 7.2.1 Targeted preferential policies to stimulate corporate innovation

In the development of the energy industry, innovation serves as the primary impetus. Therefore, it is necessary to enhance the effectiveness of R&D tax incentives and focus on the incentivizing role of tax reduction policies in fostering corporate investment in innovation. By effectively amalgamating inclusive tax reductions with structural tax reductions, one can achieve greater tax advantages. For instance, it is viable to appropriately reduce the rates of value-added tax and corporate income tax for companies operating in the green energy sector, while simultaneously augmenting the proportion of deductible R&D expenses. This measure enables companies to fully avail themselves of tax benefits. Apart from direct incentives, indirect incentives such as pre-tax deductions can be implemented to bolster the green technological innovation performance of publicly listed green energy enterprises. This approach ensures the generation of corporate innovation, facilitates breakthroughs and advancements in pivotal technologies, and stimulates the innovative vitality of corporations.

#### 7.2.2 Tailor-made differential tax policies based on local conditions

China’s vast territory encompasses diverse regions with varying resource endowments, energy distribution, and climate conditions. The social and economic development situations, as well as the emphasis on energy development, exhibit notable disparities. For instance, the northwest region possesses abundant solar resources, whereas wind and marine energy are predominantly found in the southeast coastal areas. The northeast and North China exhibit substantial potential for renewable energy development. Regional disparities in economic development levels and the business environment further influence the sensitivity of different regions to tax reduction policies. Consequently, the formulation of tax reduction policies should be customized to suit local conditions, accounting for variations in energy distribution and the regional impact of policies. Addressing the imbalanced development among enterprises across regions is crucial, necessitating the implementation of targeted tax reduction measures to mitigate the tax disparities faced by green energy companies due to regional differences.

#### 7.2.3 Balancing the short-term and long-term effects of tax policies and giving play to fiscal measures

This study demonstrates that tax reduction policies have the potential to enhance the immediate profitability of green energy enterprises, albeit with a diminishing impact over time. Simultaneously, the enduring objective of tax reduction policies is to foster high-quality advancement and attain economic transformation and upgrading. Nevertheless, protracted tax reduction policies may impose an additional burden on public finances. Consequently, it is critical to clarify the objectives and ramifications of these policies and carefully consider both short-term and long-term consequences when formulating and implementing them. In light of fiscal constraints, the government should further fortify fiscal support and guidance, facilitate the provision of financial subsidies to green energy enterprises engaged in China’s carbon peak and carbon neutrality endeavors, curtail their operational expenditures, and ensure the sustained, robust, and steady growth of the green energy sector.

#### 7.2.4 Providing on-demand financial and tax services to ensure policy implementation

China has enacted a series of tax and fee-preferential measures to bolster environmentally sustainable progress. In line with the "Opinions of the CPC Central Committee and the State Council on Fully Implementing the New Development Concept and Promoting Carbon Peak and Carbon Neutrality," various provinces have also introduced corresponding measures since October 2021. Nevertheless, the implementation process has encountered obstacles, including untimely dissemination of applicable tax incentives to companies and uncertainties surrounding subsidy amounts, methodologies, and eligibility criteria. Consequently, the efficacy of these policies has been compromised. To tackle these challenges, pertinent governmental entities should leverage both online and offline platforms to enhance policy promotion. The utilization of AI-powered analysis can furnish tailored information on available preferential policies to enterprises, address common inquiries, and ensure the effective implementation of policies.
